# Actionable pharmacogenetic markers for prediction and prognosis in breast cancer

**DOI:** 10.1186/s13167-015-0037-z

**Published:** 2015-07-22

**Authors:** Keith Sacco, Godfrey Grech

**Affiliations:** Department of Pathology, University of Malta, Msida, MSD2090 Malta

**Keywords:** Pharmacogenetics, Breast cancer, Predictive preventive personalized medicine, Therapy resistance, Tamoxifen, Aromatase inhibitor, Trastuzumab, Genotypes

## Abstract

Breast cancer is a heterogeneous disease that necessitates proper patient classification to direct surgery, pharmacotherapy, and radiotherapy. Despite patients within the same subgroup receiving similar pharmacotherapy, substantial variation in clinical outcomes is observed. Pharmacogenetic variations with direct effect on pharmacokinetics and pharmacodynamics play a central role in clinical outcomes. Pharmacogenetic markers associated with clinical outcome are known as biomarkers. They are termed prognostic biomarkers when their presence is associated with a specific clinical outcome. If the presence of such biomarkers guides treatment, they are termed predictive biomarkers. A number of pharmacogenetic markers have been described in relation to breast cancer pharmacotherapy both in the adjuvant and neoadjuvant setting. CYP2D6 allelic variants produce variable rates of tamoxifen metabolism and are associated with survival outcomes. Other biomarkers have been described in relation to other forms of endocrine therapy and trastuzumab. In neoadjuvant and adjuvant breast cancer chemotherapy, specific biomarkers were correlated with clinical outcomes and risk of drug toxicity. This review highlights key biomarkers in breast cancer pharmacotherapy with the potential of translating such study outcomes into clinical practice.

## Review

Breast cancer is the commonest female cancer diagnosed in the USA and account for one in three female cancers [1]. Such cancer patients are managed in multidisciplinary teams that coordinate surgical treatment together with administration of chemotherapy, hormonal therapy, or immunotherapy. Classification of breast tumors plays a critical role in this regard; primarily as different subtypes are associated with a specific prognosis and clinical outcome. Moreover, proper patient classification enables optimization of treatment to a specific patient.

Breast histology and tumor grade and stage have been classically used to classify breast tumors. With the advent of improved laboratory techniques, molecular receptor status of tumors has become empirical in breast cancer classification. Routine diagnostic testing for receptors in breast cancer includes the estrogen (ER), progesterone (PR), and human epidermal growth factor receptor-2 (HER-2) [2]. The aforementioned receptors are markers of prognostic outcome and guide pharmacologic treatment. Being prognostic biomarkers, their presence or absence is associated with clinical outcome irrespective of treatment [3, 4].

Estrogen positive tumors can be treated with selective estrogen receptor modulators (SERMs) such as tamoxifen or aromatase inhibitors. HER-2 positive tumors on the other hand show tumor regression in response to the monoclonal antibody trastuzumab. However, subgroup analysis of breast cancer patients showed substantial variation in clinical outcomes despite patients being on the same pharmacotherapy. This led to the hypothesis that there may be pharmacogenetic variations in patients underlying different clinical outcomes. Pharmacogenetic variations associated with pharmacokinetics and pharmacodynamics of chemotherapy, hormonal and immunotherapy, have been described. Predictive biomarkers comprise measurable traits that identify patients that are likely to benefit from treatment or exhibit adverse effects thereby facilitating a predictive response to treatment [5]. This review highlights the role of key pharmacogenetic biomarkers in breast cancer pharmacotherapy spanning endocrine, hormonal, and chemotherapy.

### Pharmacogenetic markers of tamoxifen therapy

A significant proportion of breast tumors are positive for the ER receptor. This makes the ER receptor a prognostic biomarker, since its presence is associated with a favorable prognosis. Further, as a predictive biomarker, it is an indication for prescribing hormonal therapy that interferes with estrogen signaling. Pharmacological agents disrupt estrogen signaling at various target sites hence blocking tumor cell proliferation. Drugs affecting the estrogen signaling pathway include SERMs such as tamoxifen and raloxifene. Aromatase inhibitors (AIs) are the other major class in this category with third-generation AIs including exemestane, letrozole, and anastrozole. All of these drugs have specific pharmacokinetic properties and thus vary in their distribution, metabolism, and excretion pathways, depending on activity of metabolizers and efficiency of transporter proteins that are susceptible to genetic variation. Therefore, genetic variations affecting drug pharmacokinetics serve as valuable predictive biomarkers to select patients for maximizing therapeutic effect and minimizing side-effects [6].

Tamoxifen is the most widely prescribed SERM both in locally advanced and metastatic ER positive breast cancer patients [7]. Tamoxifen is a weak anti-estrogen metabolized via CYP2D6 in the liver to produce endoxifen; a metabolite of greater potency. CYP2D6 is a polymorphic gene with more than 100 reported allelic variants, often due to single nucleotide polymorphisms (SNPs) [8]. A number of CYP2D6 alleles have been associated with either increased or decreased enzyme activity. While CYP2D6*1 is the wild-type allele (resulting in normal enzyme activity), *3, *4, and *5 are associated with negligible enzyme activity, and *9, *10, and *17 result in decreased enzyme activity. Depending on their CYP2D6 allelic variants, patients have been classified as extensive (EM), intermediate (IM), or poor metabolizers (PMs) [9–11].

These genomic variations were correlated with clinical recurrence in retrospective studies. In a cohort of 225 breast cancer patients receiving adjuvant tamoxifen, PMs had a shorter 2-year relapse free survival and a significantly shorter time-to-recurrence as compared to EM patients [11]. A subsequent retrospective study demonstrated higher recurrence rates in PMs and IMs of CYP2D6 [12]. This data suggest that selecting alternative drugs or tamoxifen dose adjustment would be indicated for PMs. However, as no prospective trial has yet shown adjuvant trial data, CYP2D6 genetic testing is not routinely incorporated into clinical practice. Doubling the dose of tamoxifen to 40 mg daily in Caucasian PMs and IMs of CYP2D6 showed a significant increase in endoxifen concentrations [13]. In patients of Japanese origin, a significantly higher concentration of plasma endoxifen was identified in CYP2D6 *1/*10 and *10/*10 homozygotes, receiving 30 and 40 mg daily of tamoxifen, respectively, with plasma endoxifen levels being comparable to wild-type individuals on standard dosing regimens [14].

CYP2D6 variants are more prominent across ethnic groups with non-Caucasians having a higher prevalence of decreased CYP2D6 activity [15]. Such data show how imperative it is to assess the risk-benefit ratio for each individual patient, and such ethnic differences must be considered when evaluating translational studies of genomic research from bench to bedside. Due to ethnic difference, a meta-analysis found insufficient evidence to robustly recommend CYP2D6 genotyping as a guide to tamoxifen treatment [16].

Genetic variants of enzymes involved in tamoxifen inactivation have been described. UDP-glucuronosyltransferase (UGT) and sulfotransferase 1A1 (SULT1A1) convert active tamoxifen to inactive soluble metabolites. Variations in SULT1A1 and UGT2B15 alleles are associated with an increased mortality risk in patients on tamoxifen [17]. However, there have been conflicting results with other studies reporting no significant association between such variables in efficacy [18, 19]. Other pharmacogenomic targets associated with tamoxifen metabolism have been studied (Table 1). Interestingly, ABCB1 gene variants are associated with multidrug resistance and decreased time-to-recurrence [20]. However, results have been conflicting among studies possibly owing to the differences in variations and genotype frequencies among different ethnic cohorts.

**Table 1:** Clinical effects observed with various pharmacogenetic markers in patients on tamoxifen pharmacotherapy

Biomarker	Effect	Reference
CYP2D6 poor metabolizers	Shorter relapse-free survival	[11]
	Higher recurrence rate	[12]
	Require increased tamoxifen dose	[13]
UGT2B15	Increased risk of recurrence	[17]
SULT1A1	Increased mortality risk	[17]
	Increased risk of recurrence	[17]
ABCB1	Shorter relapse-free survival	[20]

### Pharmacogenetic markers of aromatase inhibitors

While tamoxifen is converted to more active metabolites, third generation AIs are inactivated by metabolic pathways as the drugs letrozole, anastrozole, and exemestane are already in their active form. The aromatase enzyme catalyzes the conversion of androgens to estrogens and is coded by the CYP19A1 gene. The aromatase enzyme is ubiquitous in fat tissue and is responsible for most of the estrogen source in postmenopausal women. Given that the majority of breast cancer patients are estrogen-receptor positive and postmenopausal, aromatase is a significant therapy target in anti-tumor pharmacotherapy. AIs as adjuvant hormonal therapy showed significant improvement in disease-free survival and reduction in breast cancer events when compared to tamoxifen in the postmenopausal cohort [21]. However, side-effects mainly of a musculoskeletal nature are rather prevalent. The frequency and severity of such side-effects may be associated with pharmacogenetic variation and may predict improved treatment efficacy [22, 23].

As with CYP2D6 in tamoxifen metabolism, the CYP19A1 gene is the mostly studied pharmacogenomic target associated with AI efficacy and toxicity. Similar to CYP2D6 a number of gene polymorphisms have been described with substantial variation among ethnic groups [24]. The effect of genetic polymorphisms of CYP19A1 on response to AIs has produced conflicting results in studies. A cohort of patients with stage 4 disease treated with letrozole showed improved progression-free survival with a variant in an untranslated region of CYP19 [25]. However, these results were not confirmed when this variable was evaluated with other prognostic markers through multivariate analysis [26]. SNPs were found to predict improved efficacy of letrozole in neoadjuvant and adjuvant treatment of postmenopausal patients [27]. Conversely, SNPs such as a polymorphism in the 3′-UTR region of CYP19 predicted a poor response to letrozole [28]. Genome-wide association studies (GWAS) comparing toxicity of anastrozole to exemestane identified four SNPs within the TCL1A gene associated with musculoskeletal adverse events in women treated with AIs [29].

### Identifying pharmacogenetic markers of trastuzumab therapy

Trastuzumab is a humanized monoclonal antibody that binds specifically to the HER-2 receptor that is overexpressed in HER-2-enriched tumors thereby inhibiting cellular proliferation. HER-2 positive tumors are associated with a worse survival outcome compared to hormone receptor positive tumors; thus, the HER-2 receptor qualifies as a prognostic biomarker [30]. As a predictive biomarker, HER-2 positive patients qualify for trastuzumab treatment, which reduces tumor recurrence and overall mortality in combination with adjuvant chemotherapy [31–33]. Varying response to trastuzumab is observed, and tumor progression occurs in most metastatic patients. Trastuzumab induces tumor cell death through antibody dependent cell-mediated cytotoxicity (ADCC); a type II hypersensitivity reaction [34]. In metastatic HER-2 patients, the Fc gamma RIIIa-158 V/V genotype was associated with a higher rate of trastuzumab-mediated adverse events, yet a paradoxical improved progression-free survival was observed [35, 36]. However, to date, other studies failed to replicate results. The HER-2 receptor polymorphism I655V is associated with increased protein tyrosine kinase activity [37]. Its presence is associated with an aggressive phenotype and higher tumorigenicity. In addition, the I655V is associated with a higher risk of trastuzumab-induced cardiotoxicity albeit no effect on patient survival was observed [38]. To date, no suitable pharmacogenetic marker has been validated to optimize the benefit-risk ratio of patients under trastuzumab therapy [39].

### Pharmacogenomics altering outcomes of breast cancer chemotherapy

Cytotoxic systemic chemotherapy is administered in the neoadjuvant or adjuvant setting. As part of neoadjuvant treatment, the primary aim of chemotherapy is to downstage tumors rendering them operable. The adjuvant setting implies that chemotherapy is administered to a patient in remission with no clinical or radiological evidence of residual tumor. Adjuvant chemotherapy aims to improve disease-free survival and overall survival while minimizing the risk of recurrence [40]. The disadvantage of chemotherapy is the narrow therapeutic index associated with such drugs and the subsequent systemic toxicities that make them unpopular among patients. Pharmacogenetics may explain the different patient outcomes with respect to drug efficacy and toxicity [6, 41]. Genetic variants have been described associated with altered pharmacokinetics of anthracyclines, taxanes, cyclophosphamide, and capecitabine.

Doxorubicin is an anthracycline which inhibits the enzyme DNA topoisomerase II. It is commonly coadministered with cyclophosphamide and 5-fluorouracil and is associated with significant risk of cardiotoxicity. Carbonyl reductases are enzymes involved in the complex metabolism of doxorubicin. The presence of the variant carbonyl reductase 3 (CBR3) appeared as a double-edged sword in a study of Asian breast cancer patients. While CBR3 was associated with greater tumor reduction, patients experienced worse neutropenia [42]. In patients receiving epirubicin, another anthracycline, the presence of a polymorphism involved in drug conjugation (glucuronosyltransferase UGT2B7-His268T polymorphism) was associated with increased recurrence of breast cancer [43]. Polymorphisms of enzymes involved with oxidative stress have been associated with variable anthracycline-induced clinical outcomes. Superoxide dismutase (SOD2) and nitric oxide synthase (NOS3) variants have been associated with decreased disease-free survival and decreased progression-free survival, respectively [44, 45].

Capecitabine is a convenient form of chemotherapy as it can be administered in the oral form. The drug is used in stage IV disease and is the prodrug of 3-fluorouracil (5FU). The enzyme dihydropyrimidine dehydrogenase (DPD) catalyzes the rate-limiting step in the capecitabine and 5FU degradation. Patients with homozygous gene mutations for this enzyme suffer significant drug adverse reactions secondary to drug accumulation [46]. Further, splice variants of the DPD gene are associated with drug toxicity [47]. Increased drug toxicity to capecitabine was also observed in gene variants of the enzymes thymidylate synthase (TS) and methylenetetrahydrofolate reductase (MTHFR) [48].

Cyclophosphamide is a prodrug that is converted to the active metabolite aldophosphamide in the liver. This is then detoxified by aldehyde dehydrogenase 1A1 (ALDH1A1). This drug is ubiquitously prescribed in adjuvant breast cancer chemotherapy, yet there have been significant variations in serum drug levels reported although associations with clinical outcomes have been conflicting. The presence of ALDH1A1 is associated with triple-negative breast cancer which is known to confer a poor prognosis, while patients receiving neoadjuvant cyclophosphamide had worse clinical outcomes if ALDH1A1 is expressed [49, 50].

Paclitaxel inhibits microtubule disaggregation thus disrupting spindle formation and preventing cell division. Paclitaxel is metabolized in the liver by CYP2C8 [51]. The variant allele CYP2C8 *3 was associated with a higher rate of clinical remission with neoadjuvant paclitaxel treatment. However, increased rates of severe peripheral neurotoxicity were documented [52]. Increased risk of paclitaxel-associated peripheral neuropathy was also noted in patients with the FANCD2 haplotype [53].

## Conclusions

In the clinical setting, breast cancer patient treatment is commonly stratified according to tumor stage, histology, and presence or absence of the estrogen, progesterone, and HER-2 receptors, enabling targeting therapy with the aims of improving clinical outcomes while minimizing adverse events. In addition, molecular tumor profiling classifies the patients into luminal A, luminal B, and Basal subtypes. Of interest, patients within the same subgroup undergoing similar treatment show variable clinical outcomes. Genetic polymorphisms resulting in altered proteins, functionally affecting drug pharmacokinetics, have been shown to correlate with variable clinical outcomes both with respect to survival and drug toxicity. However, as yet, incorporation of pharmacogenomic data to the clinical setting has been hindered by a number of limitations. Primarily, results of pharmacogenomic studies have showed variable results. Thus, reaching consensus on associated outcomes with pharmacogenetic markers has been difficult. This may be due to cohort variations secondary to different ethnic groups or improper tumor classification. Further, some pharmacogenetic markers were associated with paradoxical outcomes; showing improved clinical efficacy and yet being associated with adverse drug toxicities. Future approaches using genome-wide associations may help identify other candidate genes as predictive biomarkers. However, to replicate results, standard patient classification should be based on well-defined breast cancer classification criteria while adopting standard clinical guidelines [54]. This would enable unmasking of confounding factors and hopefully facilitate the transition of using pharmacogenetic markers for targeted therapy in breast cancer. An integrative approach incorporating preventive and predictive biomarkers, risk factors and clinical data, would be the way forward to assisting targeting therapy providing a personalised medicine treatment (PPPM) [55, 56].
